# Effect of Error Augmentation on Brain Activation and Motor Learning of a Complex Locomotor Task

**DOI:** 10.3389/fnins.2017.00526

**Published:** 2017-09-27

**Authors:** Laura Marchal-Crespo, Lars Michels, Lukas Jaeger, Jorge López-Olóriz, Robert Riener

**Affiliations:** ^1^Sensory-Motor Systems Lab, Department of Health Sciences and Technology, Institute of Robotics and Intelligent Systems, ETH Zurich, Zurich, Switzerland; ^2^Reharobotics Group, Spinal Cord Injury Center, Balgrist University Hospital, Medical Faculty, University of Zurich, Zurich, Switzerland; ^3^Clinic of Neuroradiology, University Hospital Zurich, Zurich, Switzerland; ^4^MR-Center, University Children's Hospital Zurich, Zurich, Switzerland

**Keywords:** motor learning, error augmentation, random disturbance, error amplification, gait rehabilitation, rehabilitation robotics, MR-compatible robotics, fMRI

## Abstract

Up to date, the functional gains obtained after robot-aided gait rehabilitation training are limited. Error augmenting strategies have a great potential to enhance motor learning of simple motor tasks. However, little is known about the effect of these error modulating strategies on complex tasks, such as relearning to walk after a neurologic accident. Additionally, neuroimaging evaluation of brain regions involved in learning processes could provide valuable information on behavioral outcomes. We investigated the effect of robotic training strategies that augment errors—error amplification and random force disturbance—and training without perturbations on brain activation and motor learning of a complex locomotor task. Thirty-four healthy subjects performed the experiment with a robotic stepper (MARCOS) in a 1.5 T MR scanner. The task consisted in tracking a Lissajous figure presented on a display by coordinating the legs in a gait-like movement pattern. Behavioral results showed that training without perturbations enhanced motor learning in initially less skilled subjects, while error amplification benefited better-skilled subjects. Training with error amplification, however, hampered transfer of learning. Randomly disturbing forces induced learning and promoted transfer in all subjects, probably because the unexpected forces increased subjects' attention. Functional MRI revealed main effects of training strategy and skill level during training. A main effect of training strategy was seen in brain regions typically associated with motor control and learning, such as, the basal ganglia, cerebellum, intraparietal sulcus, and angular gyrus. Especially, random disturbance and no perturbation lead to stronger brain activation in similar brain regions than error amplification. Skill-level related effects were observed in the IPS, in parts of the superior parietal lobe (SPL), i.e., precuneus, and temporal cortex. These neuroimaging findings indicate that gait-like motor learning depends on interplay between subcortical, cerebellar, and fronto-parietal brain regions. An interesting observation was the low activation observed in the brain's reward system after training with error amplification compared to training without perturbations. Our results suggest that to enhance learning of a locomotor task, errors should be augmented based on subjects' skill level. The impacts of these strategies on motor learning, brain activation, and motivation in neurological patients need further investigation.

## Introduction

Robot-aided gait rehabilitation was developed to improve rehabilitation in patients with severe gait impairments (Behrman and Harkema, 2000; Riener et al., 2005). During robotic gait training, patients are provided with body weight support while a gait orthosis moves their legs into a correct kinematic gait pattern. It is thought that by moving the limb in ways that patients are otherwise not able to move would provide novel somatosensory stimulation that helps induce brain plasticity (Poon, 2004; Rossini and Dal Forno, 2004). Furthermore, robotic guidance might motivate repetitive and intensive practice in a safe environment (Reinkensmeyer and Housman, 2007). However, robotic guidance also appears to decrease physical effort during training (Israel et al., 2006), suggesting that robotic rehabilitation could potentially decrease recovery if it encourages patient's slacking, i.e., a decrease in effort, energy consumption, or attention during repeated movements when movement errors are small (Scheidt et al., 2000; Reinkensmeyer et al., 2009).

In fact, up to date, the functional gains obtained after robotic gait training are still limited (Dobkin and Duncan, 2012). There have been several clinical studies that have compared robotic gait training to conventional therapy—see (Pennycott et al., 2012) for a review. Results from these studies suggest that robotic-aided gait rehabilitation is especially suitable in the stroke acute phase, when patients can benefit from the higher degree of support from the robotic device. In general, the best results were found when the robot was employed in combination with conventional therapy (Husemann et al., 2007; Schwartz et al., 2009). In fact, a recent report suggested that robotic therapy combined with conventional therapy was more effective than conventional therapy alone in subacute stroke patients with greater motor impairment (Morone et al., 2011). Thereby, current rehabilitation robots might be working with suboptimal training strategies—only using a fraction of the rehabilitation potential—by not considering the subjects' individual needs.

Active subject participation is vital in order to provoke motor plasticity (Lotze et al., 2003), and is therefore, an important feature of gait training, especially in patients with lower motor impairments. In order to promote higher levels of subject participation and challenge, “challenge-based” controllers have been proposed, i.e., controllers that, unlike guiding controllers, make movement tasks more challenging or difficult (Marchal-Crespo and Reinkensmeyer, 2009). Research on motor learning has emphasized that errors are needed in order to drive motor adaptation (Emken and Reinkensmeyer, 2005; Reisman et al., 2013). Experimental evidence with healthy subjects has demonstrated that adaptive processes can be accelerated when trajectory errors are amplified using robotic forces during walking (Emken and Reinkensmeyer, 2005). In post-stroke patients, increasing subjects' legs phasing error (i.e., walking asymmetry) through a split-belt treadmill that moved each leg at a different speed resulted in a long term increase in walking symmetry (Reisman et al., 2013). Error amplification training also induced more robust aftereffects after locomotion training as compared to assistive training (Yen et al., 2012). However, augmenting errors did not always benefit motor learning. In a recent experiment with healthy subjects, training a golf putting task with augmented velocity errors had no effect on task performance and resulted in a decrease in motivation that lasted even after the error augmentation was retired (Duarte and Reinkensmeyer, 2015). A possible rationale for these inconsistent results is that the motivation decrease associated with error amplification might hamper learning.

Movement errors can also be induced using unexpected randomly-varying robotic forces that disturb subjects' movements during training. Recent research has stated that motor variability exhibited before training predicts motor learning ability (i.e., subjects with more variable movements showed faster adaptation; Wu et al., 2014). Unexpected randomly-varying feedforward forces might increase movement variability, and therefore, create an excellent framework to boost motor learning. Furthermore, error exploration is an important element to enhance learning, especially during the first stages of learning (Huberdeau et al., 2015). Unexpected forces might push subjects away from their “comfort zone,” and therefore, encourage them to explore and investigate the new motor tasks. In a motor learning experiment with healthy subjects, training with randomly-varying robotic forces resulted in better tracking skills than training without robotic assistance or training with repulsive forces proportional to errors (Lee and Choi, 2010). Furthermore, we found that adding random disturbing forces during training improved motor learning of a simple locomotion task, probably because the addition of unforeseen forces increased subjects' effort (muscle activation) and attention (Marchal-Crespo et al., 2014a,b).

A well-known motor learning theory, the Challenge Point Theory states that learning is maximized when the task difficulty is appropriate for the individual skill level of the performer (Guadagnoli and Lee, 2004). This is line with recent studies that found that robotic guidance seems to be especially helpful to train subjects with initial lower skill level (Marchal-Crespo et al., 2010, 2013), while error amplification was found to be more beneficial to train more skilled participants (Milot et al., 2010; Duarte and Reinkensmeyer, 2015). Additionally, error-augmenting strategies might be more suitable to enhance learning of especially simple tasks, i.e., tasks that can be learned in only one training session, since it might increase subjects' concentration (Marchal-Crespo et al., 2014b). On the other hand, in more challenging tasks, augmenting errors might decrease feelings of perceived satisfaction and competence and result in a decrease in motivation that might limit the effectiveness of error amplification on motor learning (Duarte and Reinkensmeyer, 2015).

Only few motor learning studies have compared the effectiveness of different robotic training strategies, and their relative benefits compared to unassisted practice, on motor learning. Most of these studies were performed with the upper limbs and/or using simple tasks, i.e., artificial tasks that have only one degree of freedom and can be learned in only one training session (Wulf and Shea, 2002). However, it has been shown that “*principles derived from the study of simple skills do not always generalize to complex skill learning*,” such as, relearning how to walk after a neurologic accident (Wulf and Shea, 2002). The goal of robotic therapy is to develop robotic devices that promote motor recovery, i.e., that provoke participant's motor plasticity. Currently, however, there is not a solid scientific understanding of how this goal can best be achieved. Recent work has highlighted the relevance of motor learning principles in stroke recovery and neurorehabilitation (Krakauer, 2006; Shumway-Cook and Woollacott, 2007). In fact, it has been proposed that recovery after a brain injury is a form of motor learning or relearning (Dietz and Ward, 2015). Therefore, understanding the underlying mechanisms of motor learning might suggest novel training strategies to improve neurorehabilitation. Neuroimaging evaluation of brain areas involved in learning under different robotic training strategies can provide valuable insights on the observed behavioral outcomes. Furthermore, evaluation of brain areas involved in learning might also allow tailoring the best motor training strategies to the different patterns of brain damage (Burke and Cramer, 2013).

In this study, we present results of a motor learning experiment performed with thirty-four healthy subjects to evaluate the impact of three different training strategies on motor learning of a complex locomotor task: No perturbation, error amplification, and random force disturbance. The experiment was conducted while performing functional Magnetic Resonance Imaging (fMRI) employing an MRI-compatible robotic device (MARCOS). We hypothesized that training with the challenge-based strategies would result in better motor learning in initially more skilled subjects. We also expected that training with challenge-based strategies would hamper motor learning in initially less skilled subjects. To our knowledge, no studies have evaluated the brain regions activated during entire gait-like movements. Therefore, our hypothesis related to brain activation derives from studies that investigated isolated movements of ankle and knee joints (Luft et al., 2002) or imagination of walking (Miyai et al., 2003; Jahn et al., 2004; la Fougère et al., 2010). We hypothesize to find activity in somatosensory/motor related areas (S1/M1) and supplementary and pre-supplementary motor areas (SMA/pSMA). Hypothetically, during training with the challenge-based strategies, we expect more activity within all somatosensory/motor related areas, as well as in brain areas involved in error processing, such as, the anterior cingulate cortex (Mars et al., 2005), posterior medial frontal cortex (Hester et al., 2008), and cerebellum (Tseng et al., 2007; Grafton et al., 2008). Activation in the brainstem is also expected, based on animal studies.

## Methods

### MARCOS

**Figure 1:**
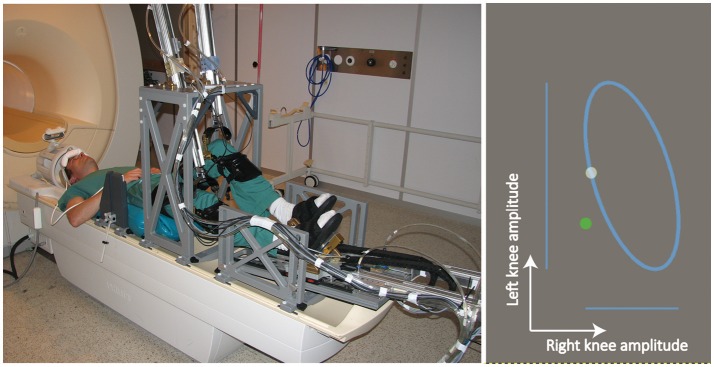
**(Left)** The MRI-compatible robotic stepping actuator MARCOS in the 1.5 Tesla MR scanner (Hollnagel et al., 2011). The participant in this figure consented to the publication of his image. **(Right)** The Lissajous figure visually presented to participants. Subjects were requested to track a white dot that moved on top of a Lissajous figure by coordinating the legs in a predefined gait-like pattern. Subjects tracked the white dot with a green dot that moved up and down when the left leg moved up and down, and moved right and left when the right leg moved up and down.

MARCOS was employed to conduct the experiment. MARCOS is an MRI-compatible robotic device pneumatically actuated and with one degree-of-freedom per leg (Hollnagel et al., 2011; Figure 1, left). MARCOS was built by the SMS-lab at ETH Zurich with low magnetic susceptibility materials to allow the assessment of brain activation using fMRI during gait-like stepping movements (Jaeger et al., 2014). The robot is actuated by two pneumatic cylinders (per leg), one attached to the subject's knee through a knee orthosis that can move the knee up and down, and a second one attached to the subject's foot sole using a special shoe, which can render forces that mimic ground reaction forces. The device allows hip, knee, and ankle flexion and extension movements in the sagittal plane that resemble on-the-spot stepping. The robot incorporates force sensors mounted at the orthoses-human contact points to measure the interaction forces between human and robot. The position of each cylinder piston is measured redundantly by optical encoders with a ceramic scale and a foil potentiometer. For more detailed information about the robot design, the reader is referred to (Hollnagel et al., 2011).

### The complex locomotor task

The experimental task consisted in tracking a white dot that moved on top of a Lissajous figure presented on a visual display (Figure 1, right) by coordinating the legs in a predefined gait-like pattern. The knees vertical displacements were mapped into the movement of a green dot on the visual display: The green dot moved up and down when the left leg moved up and down, and moved right and left when the right leg moved up and down. The predefined gait-like pattern to be learned consisted of moving the knees up and down following sinusoidal movements of equal frequency (0.5 Hz), but different amplitudes (left leg: 0.16 m; right leg: 0.08 m, i.e., axis ratio of 2) and with a phase difference between legs of 60°. This task was selected because it was challenging enough to observe learning in most of the subjects (Marchal-Crespo et al., 2014b). This task is also appealing because it resembles the abnormal gait pattern observed in stroke survivors with a paretic lower limb: An asymmetric pattern with the paretic leg performing shorter and faster steps.

### Training strategies

Subjects trained the gait-like task with one of three different training strategies: (i) No perturbation (NP): no disturbances were presented, (ii) Error amplification (EA): errors were amplified with repulsive forces proportional to errors, and (iii) Random force disturbance (RD): errors were induced with unexpected randomly-varying force disturbances. The design and evaluation of the training strategies was described in detail in (Marchal-Crespo et al., 2014b). Here, only a brief summary is given for completeness.

#### No perturbation

When training with no perturbation, subjects are free to move without feeling any disturbance or assistance force from the robot. The control approach for the no perturbation strategy is a closed-loop zero force controller that minimizes the measured interaction forces between subjects and robot. The controller includes the compensation of the weight of the knee orthosis and the dependency of pressure build-up on chamber volume (Hollnagel et al., 2013; Marchal-Crespo et al., 2014b).

#### Error amplification

In order to amplify the tracking errors (i.e., the differences between the desired and measured knee positions) created when trying to track the Lissajous figure, a proportional controller with negative impedance gain was developed. Therefore, the repulsive force applied by the knee cylinder is proportional to the tracking error, i.e., the force is smaller as smaller is the tracking error and increases proportional to the error. The forces from the error amplification controller were fed into a close-loop force controller. We saturated the magnitude of the error-amplification forces to guarantee the subjects' safety and to limit the difficulty of the task (Marchal-Crespo et al., 2014b).

#### Random force disturbance

The idea behind the random force disturbance strategy is to push subjects away from their “comfort zone,” by forcing them to experience errors/movements that would otherwise not been created. The controller applies unpredictable random perturbing forces using the knee cylinder while subjects train the tracking task. The knee cylinder applies a disturbing force that last for 0.1 s with a random magnitude between ± 100 N, every 0.5 s. The random force disturbance controller works on top of a closed-loop force controller, therefore, the subjects are always in charge of the movement generation (Marchal-Crespo et al., 2014b).

### Experimental protocol

The study was approved by the local ethical committee (Kantonale Ethikkommission Zürich, Application Number: EK-856) and conducted in compliance with the Declaration of Helsinki. Thirty-four healthy subjects (23 male), 26.6 ± 3.5 years old, gave written consent to participate. All subjects were right footed (evaluated with the Waterloo Handedness Questionnaire, (Bryden, 1977). FMRI was recorded in the MR-Center of the University of Zurich and ETH Zurich, on a Philips Achieva (Philips Medical System, Best, The Netherlands) 1.5 T MR system equipped with an 8-channel head coil.

Subjects were supine positioned with their knees fixed to the MARCOS knee orthosis, while the feet were placed in special shoes and fixed with Velcro fasteners (Figure 1, left). Head motion was minimized through several solutions, such as, custom made hip-fixations and shoulder belts, a vacuum pillow at the participants' back, and an inflatable headgear (Crania, www.pearltec.ch; Hollnagel et al., 2011). The video display of the game was projected onto a screen placed in front of the scanner and viewed by the subjects through a mirror mounted on the MRI head coil (Figure 1, left).

**Figure 2:**
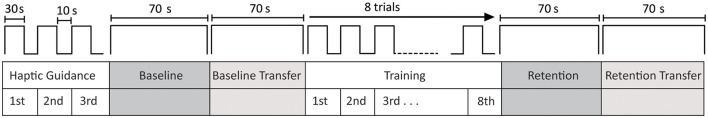
Experimental protocol. Haptic guidance was employed to help subjects to understand the locomotor task. Three trials of 30 s with the robot passively moving the subjects' legs were employed to present the locomotor task. During baseline, subjects actively tracked the Lissajous figure during 70 s in no perturbation mode. Subjects performed a second baseline test (baseline-transfer), where they tracked a similar figure but with the left leg with the smallest amplitude. During training, subjects played with no perturbation, error amplification or random force disturbance, depending on their training group. Each training session consisted of eight trials of 30 s of movement with 10 s rest between trials. The short-term retention and transfer tests followed the same structure and order as baseline and baseline-transfer tests. Subjects were scanned by the fMRI during all phases.

A parallel design was used in order to evaluate the effects of training with the three different training strategies (Figure 2). The first 23 subjects were randomly assigned to one of the three training groups: No perturbation (NP), error amplification (EA), random disturbance (RD). After a preliminary evaluation of the data, we found that the tracking errors created during baseline (i.e., before training) had a significant effect on the benefits of practicing with the different training strategies (Marchal-Crespo et al., 2014b). Although it is expected that by randomizing subjects into the different training groups would result in a balanced level of tracking error across groups, it is still possible—especially in relative small sampling sizes—to end up with imbalanced groups that could bias our results. Therefore, we decided to allocate the remainder 11 subjects to one of the three training groups using adaptive randomization methods. The idea was to yield training groups whose subjects' initial errors followed normal distributions with similar means and standard distributions. To accomplish this goal, we assigned new subjects to one of the three training groups based in the visualization of the histograms of the errors created by the subjects evaluated till the moment and the error performed by the new subjects during baseline. Eleven subjects ended in the no-perturbation group, eleven in the error-amplification group, and twelve in the random-disturbance group.

In order to instruct subjects about the task to be performed, they were presented with a video, outside the scanner room, that showed a subject in MARCOS moving alternatively his legs up and down and how these knee movements controlled the movement of a green dot on a screen. They were not informed about the training group they were assigned to, but were informed that during practice the robot could disturb them. The experiment started with a haptic demonstration phase, where the robot passively guided the subjects' legs in the desired gait-like pattern during three trials of 30 s, with 10 s of rest between trials in order to help subjects to understand the task to be performed (Hollnagel et al., 2013). During the haptic-guidance condition, subjects were instructed to relax and keep both legs passive while they observed the white and green dot moving on top of the Lissajous figure on the screen. After the haptic demonstration of the locomotor task, subjects performed the baseline test during 70 s. They were instructed to coordinate their legs in order to track the white dot that moved on top of the Lissajous figure in no perturbation mode. Transfer of learning, i.e., the capacity to apply an acquired skill on a task to another very similar task (Schmidt and Lee, 2010) is a crucial aspect of motor learning. Therefore, after baseline, subjects performed a second baseline test during 70 s (baseline-transfer), where they followed a similar Lissajous figure but with the left leg moving with the smallest amplitude (left leg: 0.08 m; right leg: 0.16 m). During training, subjects played without perturbation, error amplification or random force disturbance, depending on their training strategy group. Each training session consisted of eight trials of 30 s of movement with 10 s rest between trials. The challenge-based training strategies were applied to the left leg only, while the right leg was controlled in no perturbation mode in order to limit the task difficulty. The short-term retention and short-term retention transfer tests were 70 s long each and were performed in no perturbation mode. Overall, the experiment was <1 h. Subjects were actively scanned by the fMRI through the duration of the experiment.

### Data processing and statistical analysis

#### Behavioral data

For each protocol test and training trial, we calculated the mean tracking error for each leg as the mean of the absolute value of the difference between the measured and target knee positions. We evaluated whether the challenge-based training strategies worked as expected (i.e., they increased the error during training): We compared the error of the left leg in the first training trial to the error during baseline using a repeated measures ANOVA with training strategy as a between subjects factor. To determine whether subjects increased the left leg error when training started, a paired *t*-test between baseline and the first training trial was performed per each training group. We further compared the tracking error during the eight training trials between training groups using ANOVAs. In order to determine whether subjects adapted to the challenged-based strategies during training—i.e., they reduced the error of the left leg during training—we performed a paired *t*-test between the left leg tracking error created at the first and last (eighth) training trials.

The absolute tracking error during baseline was employed as a qualitative measure of initial skill level (i.e. the larger the error during baseline, the initially less skilled a subject was). We used K-means cluster analysis to divide subjects into two skill-based groups, based on the tracking error created during baseline. Thirteen subjects who performed systematically worse during baseline (cluster center = 0.065 m) were assigned into the novice group (5 NP, 4 RD, 4 EA), and the remainder 21 subjects were classified (cluster center = 0.041 m) as skilled (6 NP, 8 RD, 7 EA). An ANOVA was used to evaluate whether the skill groups performed differently during baseline. We used repeated measures ANOVA to test the effect that training strategies [no perturbation (NP), error amplification (EA), and random force disturbance (RD) as fixed effect], initial skill level (novice and skilled as fixed effects) and their interaction had on the tracking error reduction from baseline to retention. In order to determine whether subjects learned the complex locomotor task, a paired *t*-test between baseline and retention was performed. To determine whether subjects in each training strategy group and skill level subgroup learned the task, a Wilcoxon test between the tracking error at baseline and retention was performed. We compared the error reduction between training groups in each skill level subgroup using Kruskal-Wallis tests. Four subjects in the skilled group (2 RD, 2 EA) who performed remarkably well during baseline (error < 0.032 m) were not considered in these Wilcoxon tests in order to avoid the negative effect of learning ceiling. To test the correlation between error reduction after the different training strategies and initial skill level, Pearson's correlation tests were performed.

We used a second performance variable in order to evaluate whether subjects learned the desired phase between legs. The period between legs at each step was calculated as the difference between the time at which the left leg is at its maximum high and the time when the right leg reaches its highest position during a step. The phase was calculated as 360° over this period. For each protocol test, the absolute mean phase error (calculated as the difference between the calculated phase and the desired one of 60°) across all steps was calculated. Data from one subject in the no-perturbation group during baseline was removed from statistical analysis, since the error reached its maximum. In order to test whether subjects learned the desired phase, a paired *t*-test between baseline and retention was performed. We used repeated measures ANOVA to test the effect that the training strategies, initial skill level and their interaction had on the phase error reduction from baseline to retention.

Transfer could not be evaluated in four subjects (1 NP, 2 RD, 1 EA), because data was not correctly recorded during baseline-transfer. Transfer was evaluated using repeated measures ANOVA to test the effect of the training strategy (NP, EA, and RD as fixed effects), initial skill level (novice and skilled) and their interaction on the tracking error reduction from baseline—transfer to retention-transfer. Paired *t*-tests between the tracking error created during baseline-transfer and retention-transfer were performed in order to evaluate whether subjects in each training group transferred the learned motor skills.

Normal distribution was checked visually using Q–Q plots. Post-hoc comparisons were performed with Tukey correction. The significance value was set to α = 0.05. Statistical analyses were performed using IBM® SPSS® Software (version 23, Chicago, IL).

#### Functional MR data

FMRI of the brain was acquired using a T2^*^-weighted, single-shot, echo planar imaging sequence (echo time = 50 ms, repetition time = 3.025 s, flip angle = 90°, SENSE factor = 1.6). A total of 35 interleaved, angulated, transversal slices covering the whole brain were acquired in each volume (FOV = 220 × 220 mm, acquisition voxel size: 2.75 × 2.8 × 3.8 mm, resliced to 1.72 × 1.72 × 3.8 mm).

FMRI data were analyzed using SPM8 (Wellcome Department of Cognitive Neurology, London, UK). Images were realigned to the mean image and normalized to standard MNI space using the EPI template provided by the Montreal Neurological Institute (MNI brain) and smoothed using an 8 mm full-width at half-maximum Gaussian kernel. The estimated realignment parameter data were filtered using the discrete cosine transform matrix filter incorporated in SPM8, to remove any linear drift. In order to avoid movement artifacts, FMRI data sets that after filtering showed a total head displacement above half voxel size in each dimension were excluded from the 1st-level statistical analysis. Contrast images of the motor task performed at different time points in the MR scanner (period: baseline, training, and retention) vs. an implicit rest (no movements) condition were calculated. FMRI data sets from 10 subjects during training, and data from one subject during the retention test were excluded from further analysis because the measured head motions were above the threshold. FMRI data from a total of 24 subjects (9 NP, 7 EA, and 8 RD) during training, and 33 (10 NP, 11 EA, 12 RD) during baseline and retention were employed at 2nd-level analysis. The 1st-level contrast images were then subject to a 2nd-level full factorial group analysis (2-way ANOVA). Here we computed main effects of initial skill level and training strategy for the contrasts: Training—rest, and retention—baseline. In case of significant main effect of strategy, we compared by *post-hoc t*-tests the following contrasts:

1. Error amplification vs. no perturbation (and vice versa)
2. Error amplification vs. random force disturbance (and vice versa)
3. No perturbation vs. random force disturbance (and vice versa)

In case of a significant main effect of skill level, we compared by unpaired *t*-tests skilled vs. novice participants (across all training strategies). We also computed strategy × initial skill-level interaction effects. However, we did not perform *post-hoc* tests on the interaction, as we had not enough subjects (*n* < 10) and, thus, lacking statistical power.

All statistical tests were thresholded at *p* ≤ 0.001 (uncorrected) and were cluster-corrected (k_E_ = 27 voxels) to achieve *p* < 0.05 corrected. This cluster threshold was based on a Monte-Carlo simulation approach using a script to estimate the average size of the random clusters that can occur in our data given a *p* ≤ 0.001 (Slotnick et al., 2003).

## Results

### Behavioral data

#### Performance during training

**Figure 3:**
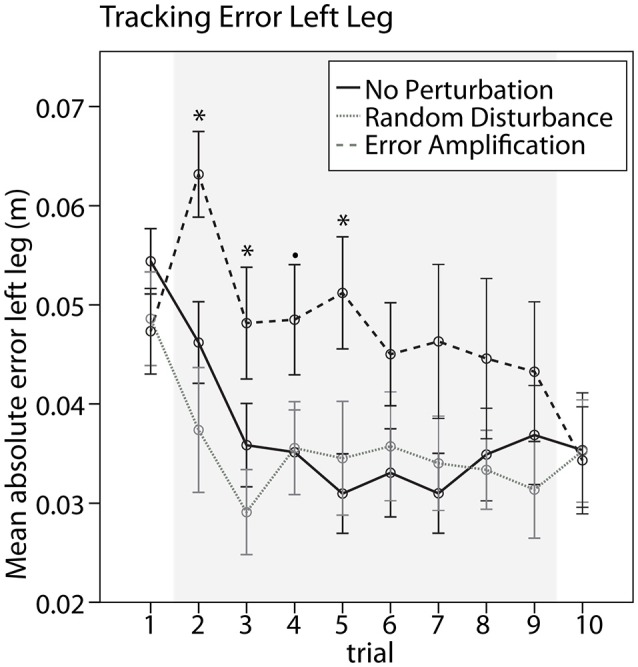
Performance during training. Mean absolute tracking error created with the left leg during baseline (trial 1), training with the different strategies (trials 2–9, shaded area), and retention (trial 10). Error bars show ± 1 CI. ^*^p < 0.05, ^•^*p* < 0.1.

The different training groups responded differently when training started, as suggested by a significant difference between training groups in the tracking error change from baseline to the first training trial [Figure 3, *F*_(2, 31)_ = 9.84, *p* < 0.001]. Subjects trained with error amplification significantly increased the error from baseline to the first training trial (*p* = 0.002), while subjects trained without perturbations and with random-disturbance did not changed the errors significantly. Subjects in the error-amplification group performed systematically worse than subjects in the random-disturbance and no-perturbation groups during the first training trials, as observed in a significant greater tracking error during the first, second and fourth training trials [Figure 3, Trial 2, *F*_(2, 31)_ = 6.62, *p* = 0.004; Trial 3, *F*_(2, 31)_ = 4.17, *p* = 0.025; Trial 4, *F*_(2, 31)_ = 2.40, *p* = 0.107; Trial 5, *F*_(2, 31)_ = 4.17, *p* = 0.025]. The differences between groups were non-significant during the last training trials. This is due to the fact that subjects in the error-amplification group adapted to the error amplification disturbance, suggested by the significant error reduction from the first to the last (eighth) training trials (paired *t*-test, *p* = 0.004). This adaptation was not observed in the no-perturbation and random-disturbance groups. Both groups reduced the error from the first to the last training trials, although not significantly. The random-disturbance and no-perturbation groups performed similarly through the duration of the training.

#### Effect of training strategies and skill level on learning

**Figure 4:**
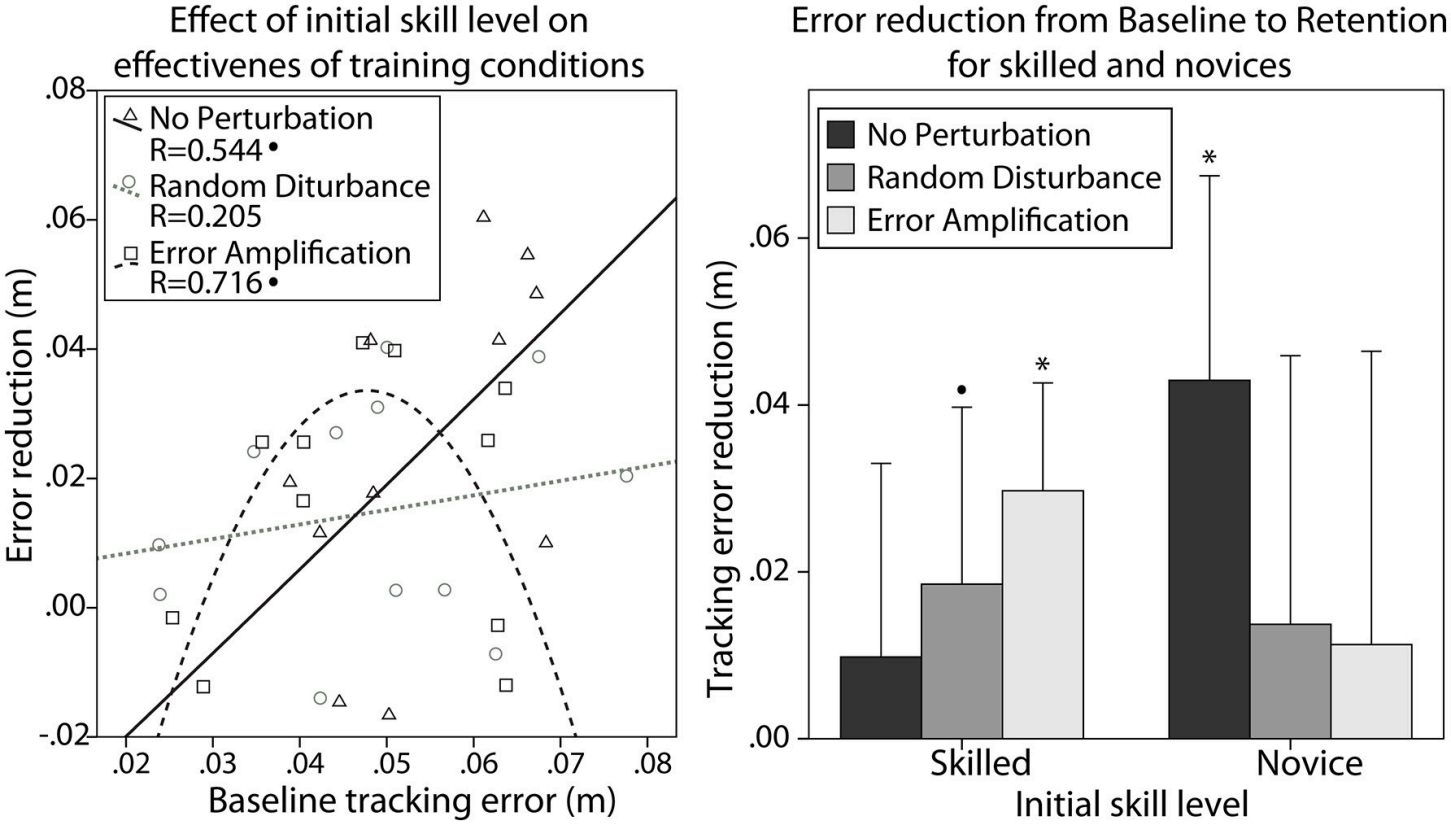
**(Left)** Effect of initial skill level (i.e., tracking error during baseline) on the error reduction from baseline to retention with the different training strategies. **(Right)** Error reduction after training with the different training strategies, in the skilled and novice groups. Error bars show ± 1 CI. ^*^p < 0.05, ^•^*p* < 0.1.

The performance during baseline was significantly different between skill groups. They showed significant differences in the tracking error during baseline [*F*_(1, 32)_ = 52.14, *p* < 0.001]. We examined the effect of the subjects' skill level (i.e., the tracking error during baseline) on the effectiveness of the different training strategies. We found a non-significant linear correlation between initial skill level and the error reduction from baseline to retention after training without perturbations (Figure 4 left, Pearson's correlation, *R* = 0.544, *p* = 0.083). We also found a quadratic relationship between the initial skill level and the error reduction from baseline to retention after training with error amplification (Figure 4 left, *R* = 0.716, *p* = 0.057).

We used repeated measures ANOVA to test the effect that different training strategies [no perturbation (NP), error amplification (EA), random disturbance (RD)], initial skill level (novice, skilled), and their interaction had on the tracking error reduction from baseline to retention. We found that all subjects reduced the tracking error after training [*F*_(1, 28)_ = 27.30, *p* < 0.001]. Subjects in all training strategies learned the task (NP: *p* = 0.011; RD: *p* = 0.016; EA: *p* = 0.022). The main effect of initial skill level on the error reduction was non-significant. The main effect of training strategy was also non-significant. However, we found an interaction between the initial skill level and the training strategy that approached statistical significance [*F*_(2, 28)_ = 3.22, *p* = 0.055]. Novices only reduced the error significantly when trained without perturbation (Figure 4 right, Wilcoxon, *p* = 0.043). In fact, novices tended to reduce the errors to a greater amount when trained without perturbation in comparison with the other training strategies (Kruskal-Wallis *p* = 0.063). The skilled group only reduced significantly the error after training with challenge-based strategies (Figure 4 right, Wilcoxon, EA: *p* = 0.043, RD: *p* = 0.075).

**Figure 5:**
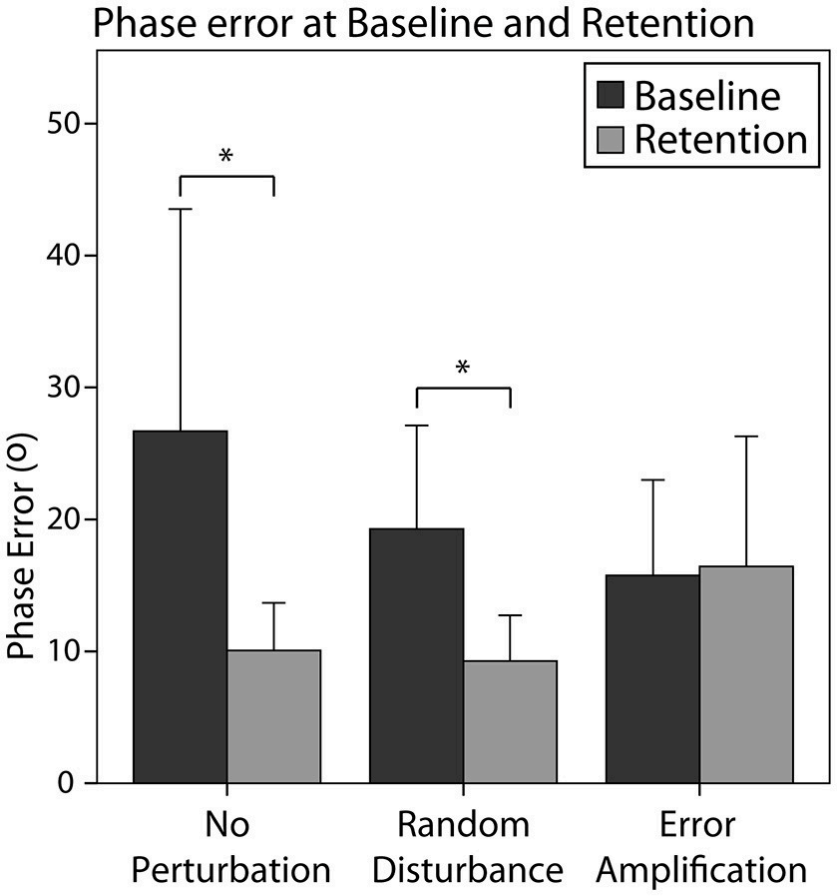
Absolute phase error for the different training groups during baseline and retention. Error bars show ± 1 CI. ^*^p < 0.05.

In general, all subjects reduced the absolute phase error from baseline to retention [*F*_(1, 28)_ = 7.50, *p* = 0.011]. The effect of the training strategy on the phase error reduction was one-sided significant [*F*_(2, 28)_ = 2.82, *p* = 0.076]. In particular, the EA group reduced the error in a smaller amount than the NP group (Figure 5, *p* = 0.077). We did not find a significant effect of skill level in the error reduction, neither an interaction effect of the training strategy and the initial skill level.

#### Effect of training strategy on transfer

**Figure 6:**
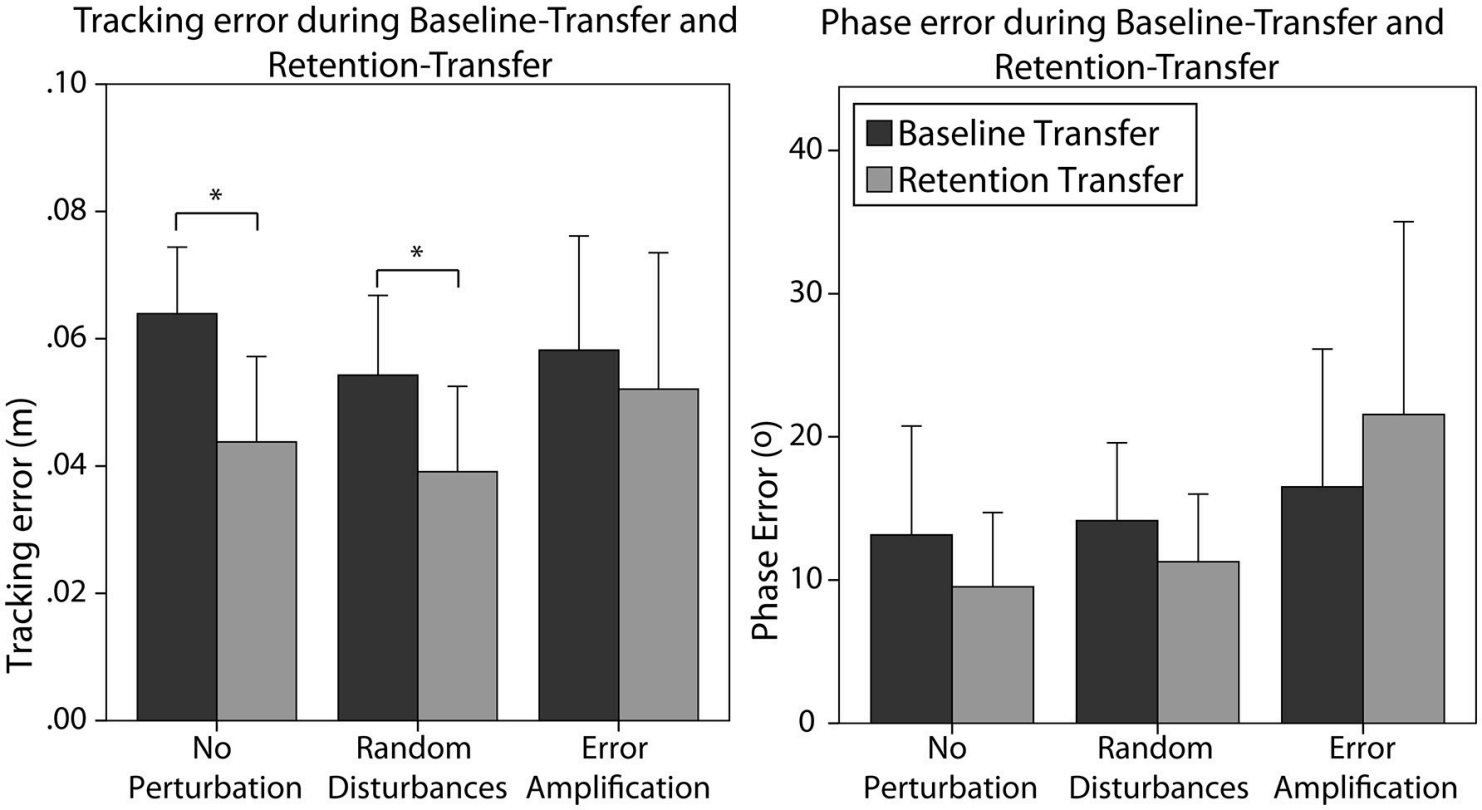
Tracking error **(Left)** and phase error **(Right)** created by the different training groups during baseline-transfer and retention-transfer tests. Error bars show ± 1 CI. ^*^p < 0.05.

Subjects generalized the learning to the untrained task, i.e., they significantly reduced the errors from baseline-transfer to retention-transfer [*F*_(1, 24)_ = 9.59, *p* = 0.005]. In particular, subjects trained without perturbation reduced significantly the tracking error (Figure 6 left, *p* = 0.016). Subjects trained with random disturbance also reduced the tracking errors significantly (Figure 6 left, *p* = 0.007). However, subjects trained with error amplification did not reduce the error from baseline-transfer to retention-transfer. The main effect of training strategy was, however, non-significant [*F*_(2, 24)_ = 1.58, *p* = 0.228]. The main effect of initial skill level was also non-significant. The interaction effect of the skill level and training strategy did not reach significance [*F*_(2, 24)_ = 2.64, *p* = 0.092].

Subjects did not significantly reduce the phase error from baseline-transfer to retention-transfer (Figure 6 right). The effect of the training strategy on the phase error reduction in the transfer task did not reach significance [*F*_(2, 24)_ = 2.97, *p* = 0.070]. As observed in Figure 6 right, subjects trained without perturbation and with random disturbance reduced the errors (although not significantly), while subjects in the error-amplification group tended to increase the errors after training. The main effect of initial skill level was non-significant. The interaction effect of the skill level and training strategy almost reached significance [*F*_(2, 24)_ = 3.25, *p* = 0.055].

### Functional MRI data

#### Training period

**Figure 7:**
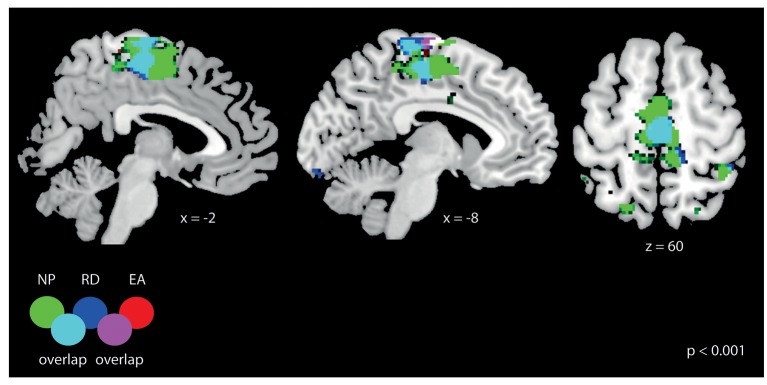
General fMRI activation for the three training strategies: NP, RD, and EA. All results are displayed at *p* < 0.001 (uncorrected, *t* > 3.6) with a cluster-extend threshold of k_E_ > 27 voxels. The activation overlaps for all three training strategies at the somatosensory cortex (at the leg area).

We first visualized the general activation for the three different strategies (NP, EA, and RD) during the training period. As it can be observed in Figure 7, all strategies lead to significant bilateral activation (*p* < 0.001, uncorrected) in the area 4a (leg area). The activation map was most widespread for NP and activation for this strategy lead also to activation in other brain regions (results not reported).

Strategy-related main effects were seen in the basal ganglia (putamen, caudate nucleus, and pallidum), thalamus as well as in different parts of the cerebellum (Table 1A). In addition, fMRI signal responses were seen in the parietal cortex such as, the intraparietal sulcus (IPS)—which represented largest cluster in the activation map—the posterior cingulate cortex (PCC) and in different regions of the visual and frontal cortex.

**Table 1:** Summary of brain activation during training for the main effect of training strategy, initial skill level, and its interaction in the training model.

**Region**	**Hemisphere**	**MNI**			**Cluster-size**
**(A) MAIN EFFECT OF TRAINING STRATEGY**					
Putamen	Left	−22	13	0	122
Middle orbital	Left	−35	47	0	225
Pallidum	Right	22	−4	0	82
Thalamus	Left	−13	−24	0	33
	Right	12	−6	12	28
Cerebellum (lobule VI)	Right	35	−41	−30	27
	Left	−20	−55	−8	52
Intraparietal sulcus (IPS)	Right	45	−55	38	934
	Left	−42	−57	39	153
Posterior cingulate cortex (PCC)	Right	1	−38	35	283
Inferior frontal gyrus (BA 44/45)	Left	−48	−13	34	326
	Right	48	23	28	45
Middle frontal gyrus	Left	−28	31	34	139
Cuneus	Right	18	−70	27	90
Amygdala	Left	−30	0	−19	61
Hippocampus	Right	40	−16	−14	59
Fusiform gyrus	Right	25	−61	5	55
**(B) MAIN EFFECT OF INITIAL SKILL**					
Medial temporal gyrus	Left	−40	14	−32	75
SPL (precuneus)	Right	−2	−66	60	56
IPS	Right	42	−49	36	64
Inferior temporal gyrus	Right	64	−38	−12	20
Rolandic operculum	Right	50	−1	13	16
**(C) INTERACTION STRATEGY** × **INITIAL SKILL**					
IPS	Right	42	−48	36	1595
	Left	−40	−57	39	320
Lingual/fusiform	Left	−20	−62	−6	226
Fusiform gyrus	Left	−37	−78	−12	28
Supplemtary motor area (SMA)	Right	2	−15	56	173
PCC	Right	4	−48	40	133
Primary motor cortex (area 4a/4p)	Right	28	−28	58	118
Middle frontal gyrus	Right	36	18	56	116
Postcental gyrus (area 1/2)	Right	24	−48	66	49
	Right	50	−40	58	109
Caudate nucleus	Right	18	12	10	108
Cerebellum (lobule VI)	Right	28	−36	−26	80
hOC3v	Left	−14	−92	2	70
Insula	Left	−26	19	0	56
Medial temporal gyrus	Right	56	−48	0	53
Superior orbital gyrus	Right	14	38	−14	47
Temporal pole	Left	−24	2	−28	44
Thalamus	Right	8	−2	12	41
Putamen	Right	16	8	4	107

*All results are reported at p < 0.001 (uncorrected, t > 3.6), F-contrast, k_E_ > 27*.

We then asked which training strategies differed in brain activation strength (Table 2). Error amplification did not lead to stronger fMRI signal responses relative to no perturbation or random disturbance. On the contrary, the contrast “no perturbations vs. error amplification” revealed numerous brain activations, which are—for simplicity—reported on *p* < 0.05 (FWE-corrected, *t* > 7.1; Table 2A). In a similar vein, the comparison “no perturbations vs. random disturbance” showed several activated clusters (*p* < 0.001, *t* > 3.6, Table 2B). The contrast “random disturbance vs. error amplification” demonstrated few significant clusters (*p* < 0.001, *t* > 3.6, Table 2C).

**Table 2:** Summary of *post-hoc t*-tests on the main effect of training strategy during training.

**Region**	**Hemisphere**	**MNI**			**Cluster-size**
(A) NP—EA^*^					
IPS	Right	43	−50	37	72
Inferior parietal lobe	Right	42	−68	39	32
Inferior frontal gyrus	Left	−57	8	31	15
Middle orbital gyrus	Left	−36	48	0	4
(B) NP—RD^**^					
Angular gyrus	Right	42	−60	30	1262
Fusiform gyrus	Left	−22	−52	−8	217
	Left	−27	−77	−5	136
Thalamus	Left	−31	−27	1	161
	Right	15	−6	12	33
Superior frontal gyrus	Left	−21	18	38	92
Ant.insula/ant. putamen	Left	−28	16	1	73
Cerebellum (lobule VI)	Left	−26	−30	−26	72
Cerebellum (dentate nucleus)	Left	−22	−52	−32	32
PCC	Right	12	−50	32	68
Lingual gyrus	Right	26	−63	4	62
Pallidum	Right	17	1	6	59
Inferior frontal gyrus	Right	47	21	27	54
Caudate nucleus	Left	−14	6	9	50
Hippocampus	Right	37	−16	−14	40
Precentral gyrus	Right	64	−3	28	36
(C) RD—EA^**^					
IPS	Right	47	−56	39	587
Inferior frontal gyrus (BA 44/45)	Left	−54	10	34	111
Superior orbital gyrus	Left	−37	52	1	37
Calcarine gyrus (V1/V2)	Right	1	−99	9	35
Superior frontal gyrus	Left	−13	53	40	34

* *p < 0.05 (FWE corrected, t > 7.1)*.

** *p < 0.001 (uncorrected, t > 3.6)*.

*The following training strategies were compared: NP—EA, NP—RD, RD—EA. For readability, results for NP—EA are shown at p < 0.05 (family-wise error corrected). All other contrasts are reported at p < 0.001 (uncorrected, t > 3.6), k_E_ > 27*.

Main effects of initial skill level were seen dominantly in right temporal and parietal regions, including the IPS (Table 1B). *Post-hoc t*-test analysis revealed that skilled subjects showed stronger activation compared to novices in the right IPS, SPL (precuneus), medial temporal lobe, and inferior temporal gyrus (Table 3). At the same threshold but with lower cluster-size cut-off (k_E_ > 15 voxels) we additionally observed activation in the brainstem and left cerebellum.

**Table 3:** Brain activation differences for “skilled vs. non-skilled” participants averaged across all learning strategies during training.

**Region**	**Hemisphere**	**MNI**			**Cluster-size**
**SKILLED vs. NON-SKILLED**					
SPL (precuneus)	Midline	0	−68	57	94
Medial temporal pole	Left	−40	14	−32	114
IPS	Right	42	−48	36	96
Inferior temporal gyrus	Right	64	−38	−12	35

*All results are reported at p < 0.001 (uncorrected, t > 3.6), F-contrast, kE > 27*.

Similar to the main effect of training strategy, strategy × initial skill-level interaction effects were most pronounced (i.e., largest activation cluster) in the IPS (Table 1C). Apart from activation in frontal and visual brain regions, we noticed further activation of the basal ganglia, primary motor cortex (M1) and somatosensory regions (e.g., SMA) as well as the cerebellum.

#### Retention—baseline period

**Figure 8:**
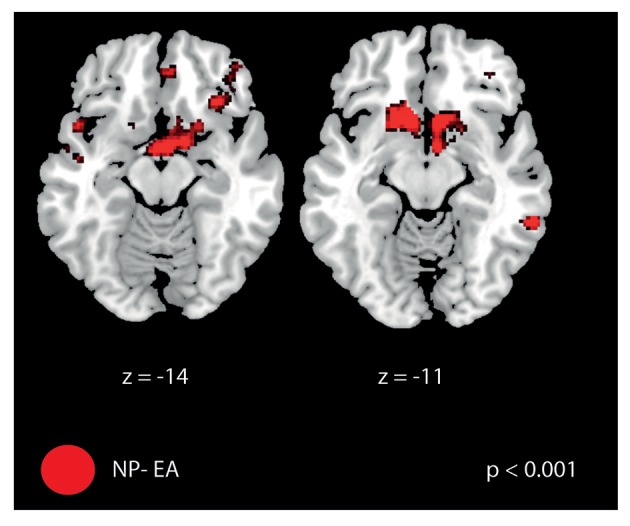
Brain activation difference between “retention—baseline” for the contrast no perturbation—error amplification. FMRI signal changes were most pronounced in the frontal cortex including the orbitofrontal cortex (for a full list of activation see Table 5).

As summarized in Table 4, a main effect of strategy was seen in the subgenual and anterior cingulate cortex and in M1. No main effect of initial skill level was seen (also not when bi-directionally comparing skilled vs. non-skilled subjects by *t*-tests) nor a strategy × initial skill-level interaction. Post-hoc analysis on the main effect of strategy revealed significant differences for the contrasts “no perturbation vs. error amplification” and “random disturbance vs. error amplification” (see Table 5). The first contrast demonstrated primarily activation in the frontal cortex. Both contrasts revealed activation in orbitofrontal regions (Figure 8).

**Table 4:** Summary of brain activation for the main effect of learning strategy for the contrast “retention—baseline”.

**Region**	**Hemisphere**	**MNI**			**Cluster-size**
**MAIN EFFECT OF STRATEGY**					
Subgenual cingulate (BA 25)	Right	2	0	13	101
	Left	−8	−2	−12	88
Anterior cingulate cortex (BA 24/32)	Left	−13	16	−8	42
Primary motor cortex (area 4p)	Right	37	−16	38	29

*Results are displayed at p < 0.001 (uncorrected, t > 3.6) with a cluster-extend threshold of k_E_ > 27 voxels. There was no significant main effect of initial skill level or an interaction effect (learning strategy × initial skill level) visible*.

**Table 5:** Summary of *post-hoc t*-tests on the main effect of learning strategy for the contrast “retention—baseline”.

**Region**	**Hemisphere**	**MNI**			**Cluster-size**
**(A) NP—EA**					
Inferior temporal gyrus	Right	62	−42	−10	41
Frontal operculum	Right	6	0	−12	617
Superior orbital gyrus	Left	−12	16	−8	
	Right	14	2	−14	
Primary motor cortex (area 3a/4p)	Right	36	−16	38	95
	Right	48	−14	38	
Anterior temporal lobe	Left	−48	10	−16	147
	Left	−54	2	−16	
	Left	−48	−6	−16	
Middle orbital gyrus	Right	22	34	−4	34
	Right	32	36	−4	
Inferior frontal gyurs/Middle orbital gyrus	Right	34	26	−16	98
	Right	44	48	−14	
	Right	32	40	−8	
Precentral gyrus (BA 44/45)	Right	56	4	36	31
Frontal operculum (fo1)	Right	4	44	−14	29
Frontal operculum (fo3)	Left	−26	36	−2	27
**(B) RD—EA**					
Superior orbital gyrus	Left	−8	−2	−12	53
Superior orbital gyrus/BA 24 and BA 25	Right	6	−2	−12	33
Middle occipital gyrus (area hOC4lp)	Right	36	−86	16	34
Inferior temporal gyrus	Left	−40	−28	18	69
Supramarginal gyrus (IPL/IPS)	Left	−48	−42	34	64
OP2	Right	30	−24	22	39
OP1 (S2)	Left	−56	−30	24	58
IPL (PFm/PGa)	Left	−52	−64	42	39
	Left	−52	−54	44	
Thalamus	Right	4	−20	20	27
	Left	−4	−22	18	

*Results are displayed at p < 0.001 (uncorrected t > 3.6) with a cluster-extend threshold of k_E_ > 27 voxels. NP and RD showed stronger fMRI signal changes compared to EA*.

## Discussion

We evaluated the impact of three error-modulation robotic training strategies on brain activation and motor learning of a complex locomotor task: No perturbation, error amplification, and random force disturbance. The experimental task consisted in learning a complex locomotor task: Coordinating the legs in a particular gait–like pattern in order to track a Lissajous figure presented on a visual display. The MRI-compatible one degree-of-freedom steeper robot (MARCOS) developed in our institution was employed to conduct the experiment, while performing fMRI in a 1.5 Tesla MR scanner. Even though none of fMRI results survives a voxel threshold of *p* < 0.05 using familywise error correction, all fMRI results are presented at a widely accepted cluster-corrected *p*-value of *p* < 0.05.

### Effects of training strategies on performance during training

The error amplification training strategy worked as expected, i.e., it significantly increased the tracking error when firstly introduced. Training with error amplification resulted in larger tracking errors during the firsts training trials compared to training with random force disturbance and no perturbation, suggesting that error amplification was the most difficult training strategy. We did not find significant differences in subjects' performance when training with random disturbance and no perturbation. The forces applied during random disturbance created short and fast change in the trajectory smoothness (Marchal-Crespo et al., 2011) and thereby, maybe the overall mean tracking error was not significantly affected. This is in line with a previous study we conducted using MARCOS to train a simple locomotor task with random force disturbance: The introduction of force disturbances did not increase the mean tracking error, but increased the muscle activation, suggesting that training with random disturbances was more physically demanding (Marchal-Crespo et al., 2014b). In fact, this higher muscular activity may explain why the tracking error during training with force disturbances did not differ from the mean error created when training without perturbations: Maybe subjects were able to cancel the tracking errors using muscular effort.

Subjects adapted to the error-amplification strategy during training. This is in line with previous research on motor learning that suggested that training with error-amplification promote the formation of an internal model (Emken and Reinkensmeyer, 2005). Subjects did not significantly reduce the tracking error when training with random disturbances, probably because the disturbing forces were unpredictably applied and the formation of an internal model was not possible.

### The training strategy that enhances learning depends on subjects' skill level

We expected better motor learning when training with the challenge-based strategies in initially more skilled subjects. We also hypothesized that training with challenge-based strategies would hamper motor learning in initially less skilled subjects. Behavioral results confirmed our hypothesis: The training strategy that enhances learning depended on subjects' initial skill level. Training without perturbations benefited learning in novices, while amplifying tracking errors during training enhanced learning in initially more skilled subjects. This is in line with previous experiments that showed that error amplification seemed to be specifically beneficial for skilled subjects (Milot et al., 2010; Duarte and Reinkensmeyer, 2015). This can be explained by the Challenge Point Theory, which states that learning is maximized when the task difficulty is appropriate for the individual subject's level of expertise (Guadagnoli and Lee, 2004). Error amplification, on the other hand, hampered learning in initially less skilled subjects, maybe because it made the task to be learned too difficult and frustrating (Duarte and Reinkensmeyer, 2015). Indeed, our fMRI data showed an involvement of the brain reward system in the error-amplification group for the contrast NP—EA, which could support this theory.

In a previous experiment performed with MARCOS and similar error-challenging robotic strategies, we found contradictory results (Marchal-Crespo et al., 2014b). Training with error amplification was most suitable for initially less skilled subjects. This contradiction might be explained by the differences in the motor task to be learned. In the previous experiment, the task consisted in trying to synchronize the non-dominant leg with the dominant leg, passively moved by the robot. Therefore, it was a simple one degree-of-freedom locomotor task, compared to the complex two degree-of-freedom bipedal locomotor task employed in the current experiment. This is in line with previous research that found that “*principles derived from the study of simple skills do not always generalize to complex skill learning”* (Wulf and Shea, 2002). For less skilled subjects, error augmenting strategies might be more suitable to enhance learning of especially simple tasks, but might hamper learning of more complex tasks. Therefore, matching the training strategy to the relative trainee's skill level to perform a specific task, may provide the greatest opportunity for learning (Marchal-Crespo et al., 2015).

Interestingly, we found that for the error-amplification group, the relationship between error reduction after training and initial skill level was quadratic. Initially proficient subjects—i.e., subjects who performed particularly well during baseline—did not benefit from error amplification. This can be explained by the behavior of the controller. The perturbations of the error-amplification strategy depend on the subjects' performance—i.e., only existing errors are amplified, with higher amplification for larger errors. Thereby, proficient subjects may not have been sufficiently challenged during training, since they were making systematically small errors and thereby, error amplification failed to have a significant impact on their performance during training.

Random force disturbances seemed to benefit motor learning in all subjects, independently of their initial skill level. It is interesting to note that the random force disturbance did not increase the mean tracking error during training. The goal of the random force disturbance strategy is to push subjects away from their “comfort zone,” so they are encouraged to examine and investigate new solutions to fulfill the motor tasks by themselves. Therefore, maybe the lack of anticipation of the random disturbing forces motivated subjects to be more concentrated and attentive during training. The random-disturbance strategy was independent of the subjects' performance, thus it might increase subjects' attention during training, even if proficient subjects created very small errors. Our fMRI data support this idea, since we found stronger activation in brain areas associated with attentional control (Shulman et al., 2003; Luks et al., 2007; such as, SPL and IPS) when training with random disturbance, compared to training with error amplification. However, we did not observe differences in brain activation between training with random force disturbances and without perturbations. This might be due to some overlapping activity in the frontal cortex (see Tables 2A,B) when we compare to error amplification. Alternatively, maybe the difference in the behavioral results was due to a change in the limbs' stiffness. Training in unstable environments tend to alter the effective stiffness of the limbs through co-contraction of muscles (Franklin et al., 2007; Marchal-Crespo et al., 2014b). However, this stiffness difference did not lead do detectable brain activation differences, maybe because the muscle co-contraction was not strong enough (Marchal-Crespo et al., 2014b).

### Error amplification hampered transfer of learning

We confirmed our hypothesis that the training strategy that enhances learning of a complex locomotor task depends on subjects' initial skill level. However, we also found that training with error amplification limited transfer of learning, while training with no perturbation and random disturbance seemed to transfer the learning gains to a similar task. Although there was no statistical proof that the transfer was greater after training without perturbation and random noise relative to error amplification, our results suggest evidence for transfer in these conditions only. The lack of transfer observed in the error-amplification group contradicts motor learning research that found a positive effect of error amplification on transfer (Milot et al., 2010).

A possible rationale for the differences observed in transfer between the challenge-based training strategies is that subjects trained with error amplification focused mainly in reducing the tracking errors to reduce the perturbing forces, and failed to interiorized or consciously understand the desired gait-like pattern. This idea is supported by the fact that subjects trained with error amplification failed to learn the correct phase difference between legs also in the trained task, while subjects trained without perturbation and random disturbance did learn the correct diphase. Therefore, the good performance of subjects after training with error amplification might have resulted from implicit learning, i.e., subjects learned without awareness of what has been learned. On the other hand, subjects trained with random force disturbance (and without perturbation) might have experienced explicit learning, i.e. they actively searched for the gait-like pattern to correctly track the presented figure. Several studies have shown that implicit learning shows negative transfer of the acquired motor skills, whereas explicit learning showed strong positive transfer (Lee and Vakoch, 1996). Therefore, based on the transfer and phase error results, we hypothesized that for the specific complex locomotion task presented in this paper, error amplification might have promoted implicit motor learning, while random force disturbance promoted explicit motor learning. Results from fMRI data support this hypothesis, since training with random disturbance revealed stronger activation in brain areas associated with explicit learning (such as, precuneus) when compared to training with error amplification (Yang and Li, 2012).

### Effects of training strategies and initial skill level on brain activation during training

During the training period, strategy and skill-level dependent effects were observed in the IPS. Human fMRI studies support a decrease in activation in pre-SMA and dorsolateral prefrontal cortex (DLPFC), but an increase in activation in the parietal lobe (especially in the IPS) as visuomotor sequence learning progresses (Hikosaka et al., 1998; Sakai et al., 1998). Grafton et al. (1998) reported that activation in the parietal lobe progressively increased with learning, whereas other groups (Seitz et al., 1990; Jenkins et al., 1994; van Mier et al., 1998) demonstrated stronger parietal activation during learning of a novel motor sequence compared to performance on a pre-learned sequence.

Our study revealed stronger activation of the SPL (precuneus) and the IPS in skilled subjects (across all training strategies) during a motor task using leg movements. Less skilled subjects did not show any significant (*p* < 0.001) activation at the pre-selected voxel-threshold relative to skilled participants. In fact, the no-perturbation and random-disturbance strategies lead stronger activation in these (but also other frontal, visual, and temporal) regions than error amplification. This could suggest that attentional control is an important factor for motor learning but in different ways. During an easy condition such as no perturbation, a high level of attention is required to maintain subjects focused on the task. In contrast, during random disturbance, subjects are disturbed in an unexpected manner (in contrast to error amplification) to perform the task and hence attentional control is required to minimize tracking errors during training.

We found main effects of strategy as well as strategy × initial skill interaction effects in different parts of the cerebellum. Some authors demonstrated a particular link between cerebellum activation and skill level (Jenkins et al., 1994; Seitz et al., 1994). For example, Toni and colleagues (Toni et al., 1998) showed a decrease in left cerebellar activation (but also in other brain regions) during a prolonged period of trial and error motor sequence learning. The right anterior cerebellum, however, showed greater activation during later learning than during initial learning. In addition, in our experiment, skilled participants showed stronger activation of the cerebellum (and brainstem) compared to novices but this was only seen at *p* < 0.001 with k_E_ > 15 voxels instead of k_E_ > 27 voxels, indicating an association between the strength of the activation in the cerebellum and the success of learning. The only region—again at *p* < 0.001 and k_E_ > 15 voxels—showing greater activation in untrained participants was the right rolandic operculum.

The basal ganglia are involved in the regulation of non-motor as well as motor sequences, and motor sequence learning (Vakil et al., 2000; Exner et al., 2002; Tzvi et al., 2014, 2015, 2016; Fermin et al., 2016). Although the activation of the basal ganglia for the training period in our study might reflect on-line learning processes, we cannot distinguish activation linked to attentional demands and on-line error correction (adaptation). The activation of the basal ganglia was evident for the contrast “no perturbation (NP)—random disturbance (RD).” This was a bit surprising, as we did not expect a particular involvement of the basal ganglia in a very simple task such as no perturbation compared to random disturbance and error-amplification strategies. We conclude that basal ganglia are rather involved in the learning of non-challenging task, in which the sequence of motor movements is not disturbed by noise or by any error amplification.

### Effects of training strategies on brain activation during learning

The contrast “retention—baseline” revealed activation within the frontal cortex but also in sensorimotor regions (e.g., M1, parietal opercular regions, i.e., OP1 and OP2, Young et al., 2004). This activation could reflect learning (as the error rates drop during retention relative to baseline) but it is difficult to differentiate between mechanisms related to attention and error correction. One interesting observation was the involvement of orbitofrontal regions during no perturbation relative to error amplification (Table 5, Figure 8). In fact, practicing with error amplification is related to a persistent lower self-reported level of enjoyment (Duarte and Reinkensmeyer, 2015). The systematic large errors experienced during training with error amplification, which made the task more challenging, resulted in unconventionally low activation of the reward system. We computed several contrasts in order to further evaluate the effect of the skill level as we found differences comparing NP—EA in the reward system: (NP—EA skilled) > (NP—EA non-skilled) and vice versa,. We also performed the contrasts “EA skilled vs. EA non-skilled” and “NP skilled vs. NP non-skilled.” Yet, we did not observe any activation differences for these contrasts (at *p* < 0.001, uncorrected), suggesting that the reward system was not differently activated between skill groups. However, when we used an unconventionally low threshold of *p* < 0.01 (uncorrected), we found some differences in the reward system between skilled and non-skilled subjects comparing conditions. Of course, this needs to be examined in future studies on the role of affective components during motor learning. However, it is evident from other studies that affective control (resulting in high motivation) is an important factor during complicated motor learning tasks (McAuley et al., 1989; Duarte and Reinkensmeyer, 2015).

### Implications for robot-aided gait rehabilitation

Recovery after a brain injury has been proposed to be a form of motor learning or relearning (Dietz and Ward, 2015). However, we cannot guarantee that the impact on motor learning of the training strategies here investigated do not differ in neurological patients. Patients probably have a lower initial skill level and therefore, their optimal challenge point (i.e., where subjects show the best motor learning) may be at a different level. Hypothetically, the haptic guidance mode may be especially suitable for more disabled patients, as suggested in Klamroth-Marganska et al. (2014).

Based on the results presented in this paper, we are developing new training strategies to improve rehabilitation outcomes using the Lokomat (Hocoma, Switzerland)—a commercially available robotic gait trainer that comprises two actuated leg orthoses (Riener et al., 2005). We developed a novel error-modulating robotic strategy that limits large errors that can be dangerous and frustrating with haptic guidance, while amplifies task relevant small errors. We also developed a random disturbance controller that can work together with the error-modulating controller (Rüdt et al., 2016). We hypothesize that an optimal framework for motor learning and neurorehabilitation would consist on a combination of all these strategies: Random force disturbance would increase subjects' concentration on the task, error amplification would increase subjects' active participation and awareness of small task relevant errors, and haptic guidance would limit dangerous and/or discouraging errors.

We plan to perform motor learning experiments with neurological patients employing the different controllers presented here, and the novel controllers derived from the actual findings. Results from the motor learning experiments performed with neurological patients will provide an insight into motor learning in the impaired motor system and may suggest new neurorehabilitation therapies and novel ways to use robots in rehabilitation. It has been hypothesized that in order to enhance recovery after a neurologic insult it is crucial to increase the dose and intensity of therapy (Dietz and Ward, 2015). We believe that employing a training strategy that optimizes learning based on patients' specific impairments and specific motor task to be performed would be a more effective approach than just increasing dose and intensity of raw therapy.

## Conclusions

We investigated the effect of robotic training strategies that augment errors–error amplification and random force disturbance—on brain activation and learning of a complex locomotor task. We found that the most effective training strategy depends on subjects' initial skill level. Training without perturbations was especially suitable to enhance motor learning in initially less skilled subjects, while more skilled subjects benefited from error amplification. However, training with error amplification limited transfer of learning. Random disturbing forces induced learning and promoted transfer in all subjects, probably because they increased subjects' attention. A possible rationale for the differences in transfer between the challenge-based strategies is that for the specific complex locomotion task presented in this paper, error amplification might have promoted implicit motor learning, while random force disturbance promoted explicit motor learning.

FMRI analysis revealed main effects of strategy and skill level during training. These neuroimaging findings indicate that gait-like motor learning depends on interplay between subcortical, cerebellar, and fronto-parietal brain regions. An interesting observation was the low activation seen in the brain reward system after training with error amplification compared to training without perturbations.

Our results suggest that learning a complex locomotor task can be enhanced when errors are augmented based on subjects' initial skill level. The impacts of these strategies on motor learning, brain activation and motivation in neurological patients need further investigation.

## Author contributions

LMC, LJ, and RR contributed to the experimental design and project supervision. LMC, LJ, and JL participated in the study design and data acquisition. LM, LJ, and JL performed the fMRI data analysis. Interpretation of the fMRI results was performed by LM. LMC performed the behavioral data analysis and interpretation of results. LMC and LM prepared the manuscript. All authors read and approved the final manuscript.

### Conflict of interest statement

The authors declare that the research was conducted in the absence of any commercial or financial relationships that could be construed as a potential conflict of interest.
